# Biophysics of Active Vesicle Transport, an Intermediate Step That Couples Excitation and Exocytosis of Serotonin in the Neuronal Soma

**DOI:** 10.1371/journal.pone.0045454

**Published:** 2012-10-03

**Authors:** Francisco F. De-Miguel, Iván Santamaría-Holek, Paula Noguez, Carlos Bustos, Enrique Hernández-Lemus, J. Miguel Rubí

**Affiliations:** 1 Instituto de Fisiología Celular, Universidad Nacional Autónoma de México. Circuito Exterior, Ciudad Universitaria, Mexico City, México; 2 UMJ-Facultad de Ciencias, Universidad Nacional Autónoma de México Campus Juriquilla, Querétaro, México; 3 Departamento de Genómica Computacional, Instituto Nacional de Medicina Genómica, Mexico City, México; 4 Facultat de Fisica, Universitat de Barcelona, Barcelona, Spain; CNRS – Université Aix Marseille, France

## Abstract

Transmitter exocytosis from the neuronal soma is evoked by brief trains of high frequency electrical activity and continues for several minutes. Here we studied how active vesicle transport towards the plasma membrane contributes to this slow phenomenon in serotonergic leech Retzius neurons, by combining electron microscopy, the kinetics of exocytosis obtained from FM1-43 dye fluorescence as vesicles fuse with the plasma membrane, and a diffusion equation incorporating the forces of local confinement and molecular motors. Electron micrographs of neurons at rest or after stimulation with 1 Hz trains showed cytoplasmic clusters of dense core vesicles at 1.5±0.2 and 3.7±0.3 µm distances from the plasma membrane, to which they were bound through microtubule bundles. By contrast, after 20 Hz stimulation vesicle clusters were apposed to the plasma membrane, suggesting that transport was induced by electrical stimulation. Consistently, 20 Hz stimulation of cultured neurons induced spotted FM1-43 fluorescence increases with one or two slow sigmoidal kinetics, suggesting exocytosis from an equal number of vesicle clusters. These fluorescence increases were prevented by colchicine, which suggested microtubule-dependent vesicle transport. Model fitting to the fluorescence kinetics predicted that 52–951 vesicles/cluster were transported along 0.60–6.18 µm distances at average 11–95 nms^−1^ velocities. The ATP cost per vesicle fused (0.4–72.0), calculated from the ratio of the ΔG_process_/ΔG_ATP_, depended on the ratio of the traveling velocity and the number of vesicles in the cluster. Interestingly, the distance-dependence of the ATP cost per vesicle was bistable, with low energy values at 1.4 and 3.3 µm, similar to the average resting distances of the vesicle clusters, and a high energy barrier at 1.6–2.0 µm. Our study confirms that active vesicle transport is an intermediate step for somatic serotonin exocytosis by Retzius neurons and provides a quantitative method for analyzing similar phenomena in other cell types.

## Introduction

Serotonin and other molecules in the nervous system act as conventional transmitters when released from presynaptic endings or as modulators when released extrasynaptically from the soma, dendrites and axon varicosities [1]–[2]. Somatic exocytosis in central neurons of vertebrates and invertebrates is triggered by transmembrane depolarization through the mobilization of internal pools of vesicles towards the plasma membrane, with which vesicles continue to fuse even for several minutes after the end of the depolarization [3]–[5]. The distance between the resting vesicles and the plasma membrane and the long latency of exocytosis following depolarization suggest the use of an energy-dependent mechanism as an intermediate requirement for the excitation-secretion coupling, maybe similar to that in chromaffin and other secretory cells [6]–[13]. Although extrasynaptic exocytosis and its effects are being demonstrated in an increasing number of neuron types [1], [2], [14], [15], the forces and energy expenses of the vesicle transport used to reach this type of exocytosis in neurons or in excitable endocrine cells still remain unexplored. Here we studied these finely-regulated processes to understand a general aspect of exocytosis.

For our study, the large (60–80 µm diameter) soma of serotonergic leech Retzius neurons, either in the ganglion or in culture offers several advantages. Serotonin is stored in clusters of large (100 nm diameter) dense core vesicles [16]–[18] and electrical stimulation with trains of ten impulses at 20 Hz evokes exocytosis for the following 2–5 minutes, contrary to the effect of 1 Hz stimulation, which fails to evoke exocytosis [3]. Electron micrographs taken from neurons at rest or after 1 Hz stimulation contain clusters of dense core vesicles resting away from the plasma membrane, whereas after 20 Hz stimulation a large proportion of the vesicle clusters appear closely apposed to the plasma membrane [19], [20]. Since somatic secretion in Retzius neurons and in other neuron types depends on transmembrane calcium entry followed by calcium release from intracellular stores [19], [21], a plausible hypothesis is that increases of free cytoplasmic calcium trigger the transport of vesicles towards the plasma membrane through the activation of cytoskeletal-based molecular motors. This may explain, at least in part, the minute scale duration of exocytosis, which is 1–2 orders of magnitude longer than the duration of depolarization [3].

**Figure 1:**
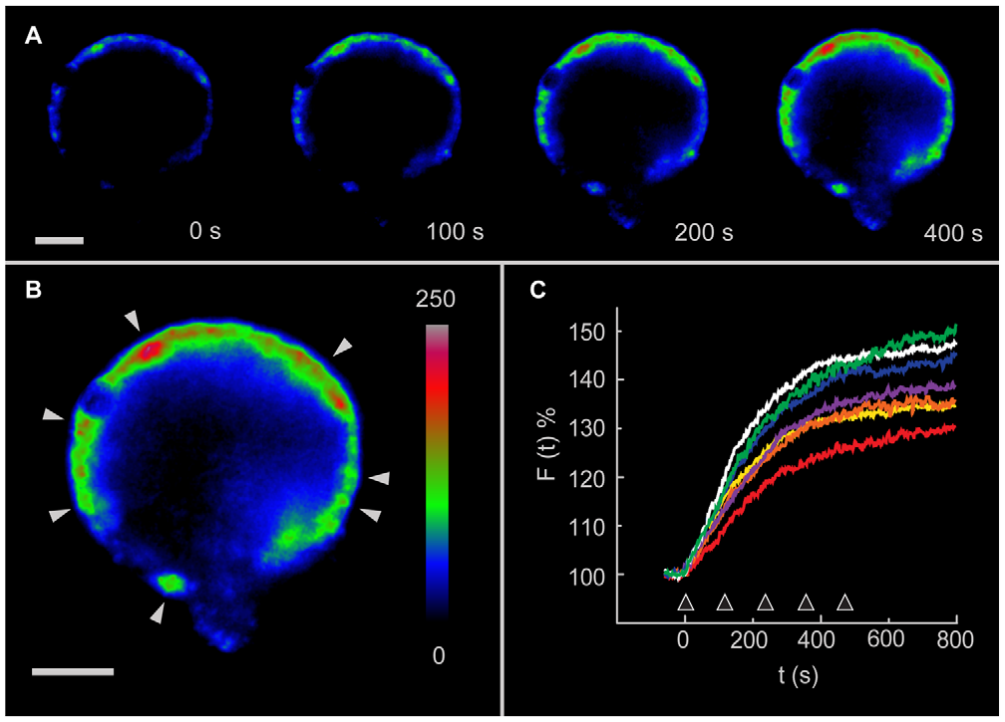
FM1-43 fluorescence increases induced by electrical stimulation. **A**. Temporal sequence of images focused on the neuronal equator showing the slow development of a transmembrane spotted fluorescence pattern in response to electrical stimulation at t = 0. The remnant segment of the primary axon points downwards. **B**. Amplification of an image of the same neuron in A 500 s after stimulation. The arrows point to the fluorescence spots in focus from which the measurements in C were made. **C**. The fluorescence kinetics of different fluorescent spots were similar. Scale bars  = 20 nm.

We used electron microscopy of neurons in the ganglion to quantify the distances between the vesicles and the membrane at rest, to search for cytoskeletal elements associating the vesicles and the plasma membrane and to estimate the densities of the vesicles in the clusters. The kinetics of exocytosis induced by intracellular trains of impulses were analyzed in cultured neurons from the fluorescence increases of the dye FM1-43 as it stained vesicles that fused with the plasma membrane [22]. The contribution of microtubules to vesicle transport was disrupted by incubation with colchicine. These data were used to feed a mathematical model based on constrained diffusion in the presence of molecular motor forces. This combined approach rendered an estimate of the number of vesicles (n_0_) transported and fused per active zone, their traveling distances (d) and velocities (v). The free energy cost of the process (ΔG) obtained from the work equations divided by the free energy of the cleavage of an ATP molecule (ΔG_ATP_) and by the number of vesicles fused per active zone rendered an estimate of the ATP expenses per vesicle fused (ATP_ves_).

**Figure 2:**
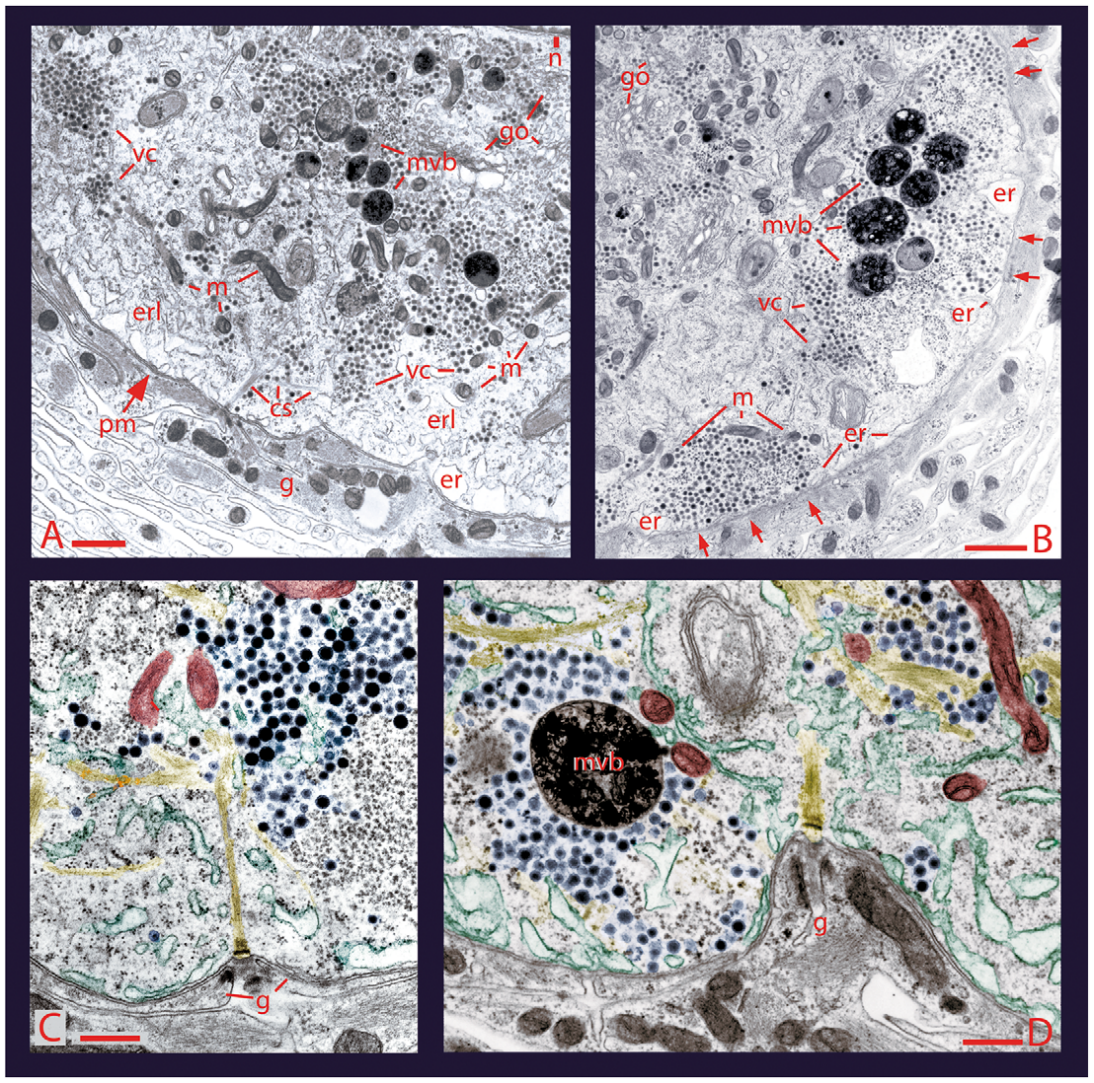
Ultrastructure of somatic active zones. **A**. Electron micrograph of a Retzius neuron in the ganglion after stimulation with 1 Hz trains, a stimulation frequency subthreshold to evoke secretion. In these conditions, the vesicle clusters (vc) remained at a distance from the plasma membrane (pm), although they were indirectly bound to it through bundles of microtubules and were near to mitochondria (m) and endoplasmic reticulum (er). The cytoplasmic space between the plasma membrane and the vesicles contained smooth endoplasmic reticulum layers. More internally, there was another population of vesicle clusters and multivesicular bodies (mvb). Retzius neurons were surrounded by layers of a giant glial cell (g). Scale bar = 500 nm. **B**. After stimulation with 20 Hz trains, the vesicle clusters were apposed to the plasma membrane (arrows) and flanked by endoplasmic reticulum and mitochondria. Scale bar  = 1 µm. **C**. Higher magnification of a pseudo-colored active zone after 1 Hz stimulation. Vesicles (blue) were at a distance from the plasma membrane, forming associations with cytoskeletal bundles (yellow), mitochondria (brown) and endoplasmic reticulum (green). The extracellular side of the anchor site of the cytoskeleton with the plasma membrane had triplets of glial cell fingers. Scale bar  = 500 nm. **D** After 20 Hz stimulation, vesicles were apposed to the membrane near the anchor sites of the cytoskeleton. Mitochondria had maintained their proximity to the vesicle clusters and endoplasmic reticulum. The presence of multivesicular bodies suggests vesicle degradation upon endocytosis. Scale bar  = 500 nm.

## Materials and Methods

### Ethics Statement

Animal research was conducted according to the statements of the Animal Committee of the Instituto de Fisiología Celular, Universidad Nacional Autónoma de Mexico.

**Figure 3:**
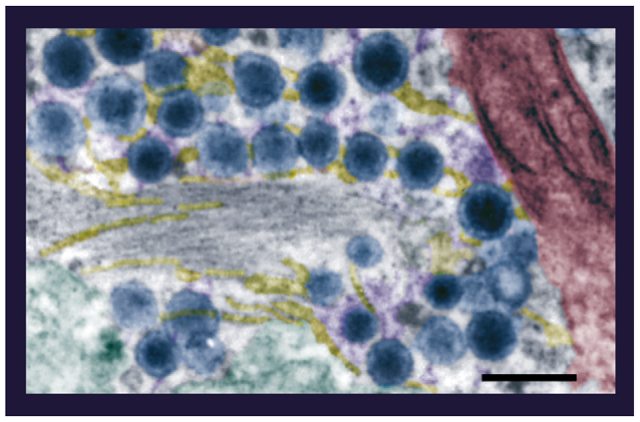
Interactions between the mobile organelles of an active zone. Higher magnification of a thick (∼120 nm) electronmicrograph containing vesicles (blue) attached to microtubules (some individuals characterized by their 20–25 nm diameters are in yellow) through different filaments (purple). Mitochondria (brown) and endoplasmic reticulum (green) were in the periphery of the clusters. Pseudocolors are as in Fig. 1. Scale bar  = 200 nm.

**Table 1:** Definition of parameters.

Symbol	Definition	Dimensions
A	Area from which the fluorescence was measured.	L^2^
a	Radius of a vesicle	L
ATP_ves_	ATP_ves_ Number of ATP molecules used per vesicle fused	
	Dimensionless constant indicating the effectiveness of the fusion of vesicles to the plasma membrane	
	Friction coefficient per mass unit.	T^−1^
D	Diffusion coefficient of vesicles in the medium.	L^2^T^−1^
b	Initial distance between the vesicle clusters and the plasma membrane.	L
β	Gibb's free energy	Joules
ΔG_ATP_	Gibb's free energy of an ATP cleavage	Joules
F_0_	Baseline value of the fluorescence intensity.	a.u.
F(t)	Fluorescence as a function of time, the indirect measure of vesicle's fusion with the plasma membrane	a.u.
F	Force	Newtons
f_i_	Sum of forces per mass unit applied on the vesicles.	LT^−2^
f_f_	Friction forces per mass unit.	LT^−2^
f_R_(t)	Random forces due to thermal agitation per mass unit.	LT^−2^
f_el_	Elastic forces per mass unit.	LT^−2^
f_mot_	Molecular motor's force per mass unit.	LT^−2^
g	Dimensionless function of the frequency	
J = ρv	Current density of vesicles.	L^−2^T^−1^
κ	Elastic constant of the cytoskeleton.	MT^−2^
k_B_	Boltzmann constant	ML^2^T^−2^K^−1^
m	Mass	M
η	Viscosity	ML^−1^T^−1^
n_0_	Number of vesicles per cluster.	
r_0,i_	Initial position of the vesicle clusters.	L
ρ	Number of parts per volume unit	L^−3^
T	Temperature	K
t	Time	T
τ_s_	Time scale at which friction and elastic force compete.	T
u	Instantaneous velocity of a vesicle cluster.	LT^−1^
v	Average velocity of vesicle clusters movement towards the plasma membrane, (with v = |v|, the magnitude of the velocity).	LT^−1^
W	Work	Joules
ω	Characteristic frequency of the elastic force.	T^−1^

### Electron microscopy

The general procedures were as in [20]. In brief, neurons in the ganglion of leeches Hirudo verbana [23] were stimulated with 10 trains of 10 impulses at frequencies of 1 or 20 Hz separated by 1 min intervals. Ganglia were then perfused with 0.08 M cacodylate buffer (pH 7.4; Sigma, St. Louis, MO), and fixed for 60 min with 0.6% glutaraldehyde and 0.4% paraformaldehyde, followed by postfixation for 60 min in 1.0% osmium tetroxide [13]. Thin (70–100 nm) sections were observed in a JEOL 1010 electron microscope (JEOL USA Inc., Peabody, MA). Electron micrographs were digitized at 1200 dpi in CMYK mode. For illustration purposes some structures were selected by use of the fast selection tool and pseudo-colored by applying the color equilibrium tool of Photoshop (Adobe, Novato CA).

**Figure 4:**
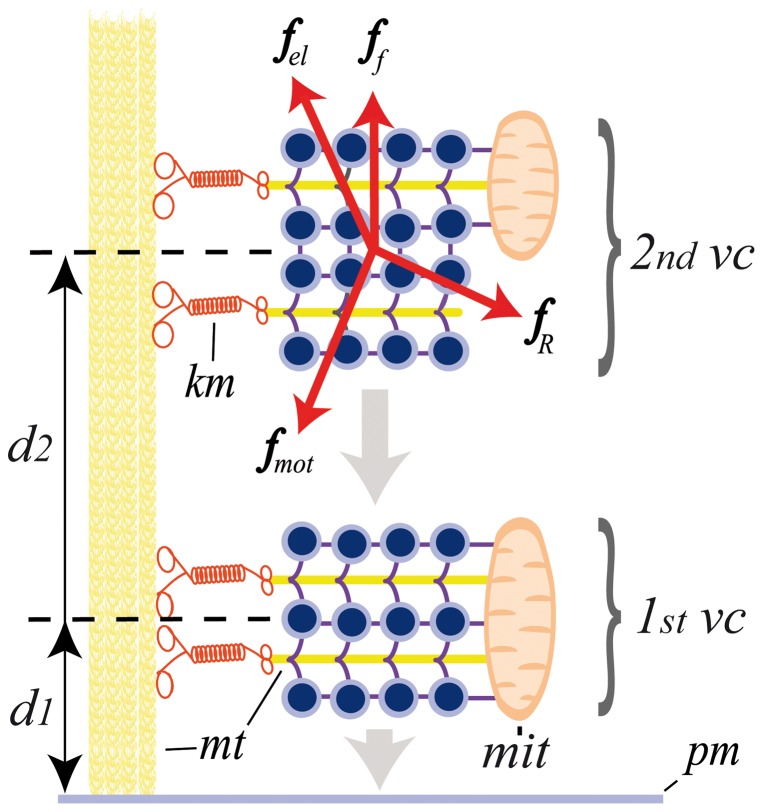
Ultrastructure of a somatic release site and the forces acting on vesicle transport. Microtubules (yellow) are the rails for the transport of vesicles clusters (vc) over long distances. Dense core vesicles (blue) are attached to microtubules (mt) through thin filaments (purple), forming clusters (vc). In response to electrical stimulation, the cluster is transported towards the plasma membrane along microtubule bundles (vertical yellow tube) trough kinesin motors (km). At rest, the vesicle clusters are at a distance (d). Four forces (red arrows) affect the active transport. These are the motor (f_mot_), viscoelastic (f_el_ and f_f_) and diffusion (f_r_) forces. Upon electrical stimulation, the traveling velocity (v = d/t) depends on the motor and elastic forces acting in combination and on the cargo imposed to the motors by the vesicle cluster and other organelles such as mitochondria (not shown). The number of vesicles that fuse is n_0_. As it approaches the plasma membrane (pm), the cluster enters the actin cortex (ac) and a second set of motors, the actin-myosin system (mym) becomes activated and contributes to the second stage of the transport.

### Isolation and culture of neurons

Retzius neurons were isolated from the central nervous system of adult leeches, as described elsewhere [24]. Experiments were performed at room temperature (25°C) after 1–8 days in culture.

**Figure 5:**
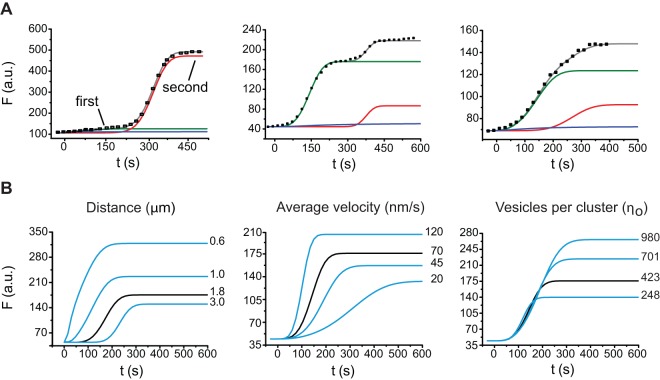
Model fittings to FM1-43 fluorescence kinetics. **A**. fluorescence kinetics of three fluorescent spots from different neurons in response to 20 Hz trains. The black symbols are the experimental data and the continuous lines are the model fittings. Two sigmoidal steps characterized the kinetics of fluorescent spots. The green and red lines are the model fittings to the first and second sigmoidal kinetics, respectively. The blue lines predict the diffusion component when the motors are eliminated by zeroing the velocity of the motors. **B**. Model simulations of the sigmoidal increase shown on the left of panel in A, when the distance (left), velocity (center) and number of vesicles per cluster (right) values were varied in the model. The black lines are the best fits to the data. The values used for the simulations are on the right of each curve. Note the different effects of each variable on the simulations.

### Stimulation of exocytosis

Exocytosis was analyzed from fluorescence increases upon the incorporation of the fluorescent dye FM 1–43 during vesicle fusion [25]. FM 1–43 (2 µM) was added to the bath and 10 minutes later neurons were impaled and hyperpolarized to −60 mV to avoid spontaneous firing. Stimulation consisted of trains of 10 action potentials produced by intracellular injection of 10 ms current pulses at 20 Hz [3], [4]. To evoke the mobilization and fusion of more internal vesicle clusters, subsequent trains were delivered at 2 minute intervals. For current injection we used borosilicate microelectrodes with resistances of 12-20 MΩ when filled with 3 M KCl. Electrical recordings were acquired by an analogue-to-digital board Digidata 1200 (Axon Instruments) at a sampling frequency of 20 KHz using pCLAMP9 software (Axon Instruments) and stored in a PC. To test the microtubule dependence of exocytosis we added colchicine (Calbiochem 10 or 100 µM) to the culture medium 30 minutes before the addition of FM1-43. Similar results were obtained with either colchicine concentration.

**Figure 6:**
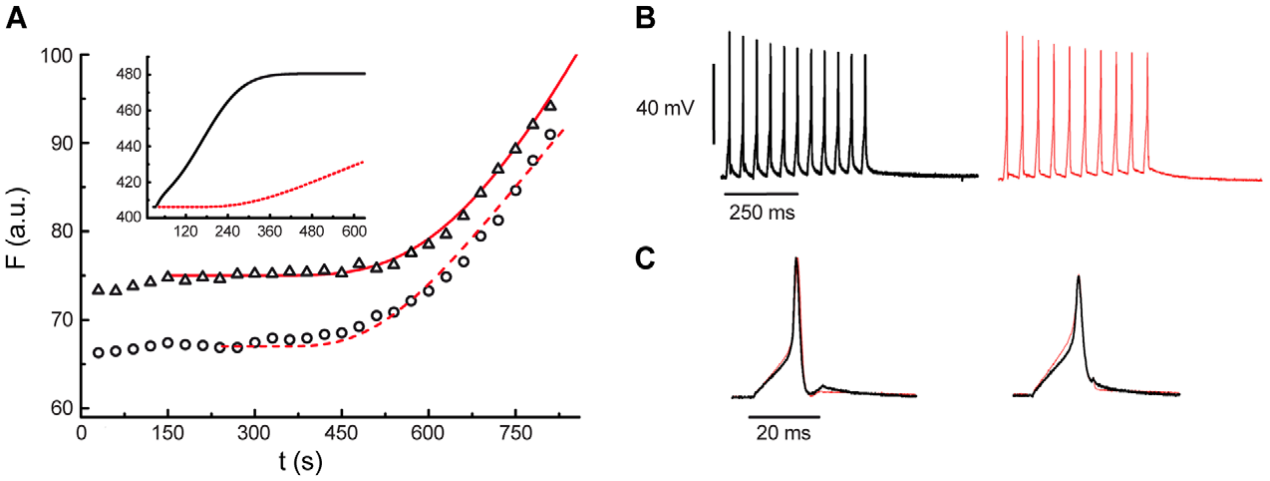
Microtubule disruption eliminates evoked FM1-43 fluorescence increases. **A**. FM1-43 fluorescence kinetics (arbitrary units) of two fluorescent spots from neurons stimulated at 20 Hz in the presence of colchicine. The fluorescence increases were smaller and slower than those produced by a single 20 Hz train in neurons stimulated in the absence of colchicine (inset). The symbols are the experimental data and the red lines are the model fittings obtained after cancelling the motor contributions in the model. **B**. Trains of action potentials produced by neurons stimulated without colchicine (black) and in the presence of 100 µM of colchicine (red). **C**. superimpositions of the first and last action potentials of the trains above show that colchicine did not affect the neuronal electrical activity.

**Table 2:** Values predicted from model fittings to the fluorescence kinetics of 9 spots from an equal number of neurons.

Neuron	Vesicles per active zone (η_0_)	K(s^−1^)	*f*_el_ (10^−3^m^2^/s^2^)	*f*_mot_ (10^−2^m^2^/s^2^)	d (µm)	υ (νμ/σ)	D (10^−2^ µm^2^/s)	ATPves
a	**95**	**31.6**	**0.7**	**1.8**	**0.6**	**95.0**	**0.06**	**55.4**
	*675*	*34.2*	*3.3*	*1.9*	*2.7*	*35.0*	*0.11*	*2.4*
	770							
b	**169**	**59.2**	**3.9**	**4.4**	**1.1**	**45.0**	**0.65**	**7.2**
	*930*	*53.2*	*12.3*	*3.5*	*4.3*	*27.5*	*0.10*	*0.4*
	1099							
c	**174**	**30.8**	**1.0**	**0.6**	**1.1**	**20.0**	**0.7**	**14.2**
	*229*	*43.6*	*4.9*	*3.4*	*2.6*	*60.0*	*0.17*	*1.1*
	403							
d	**72**	**34.6**	**1.4**	**1.1**	**1.05**	**15.0**	**0.06**	**12.5**
	*641*	*33.9*	*2.6*	*2.7*	*2.25*	*60.0*	*0.28*	*6.0*
	713							
e	**952**	**31.9**	**1.4**	**2.2**	**1.4**	**50.0**	**0.15**	**3.4**
	*125*	*46.9*	*13.7*	*3.2*	*6.2*	*25.0*	*0.08*	*7.6*
	1077							
f	**95**	**31.6**	**0.6**	**2.2**	**0.6**	**70.0**	**0.09**	**73**
	*262*	*46.9*	*9.0*	*2.0*	*4.1*	*11.5*	*0.06*	*2.4*
	357							
g	**423**	**41.8**	**3.1**	**3.5**	**1.8**	**70.5**	**0.25**	**9.9**
	*52*	*62.8*	*24.2*	*4.8*	*6.1*	*26.0*	*0.10*	*16.5*
	475							
h	**260**	**56.6**	**4.1**	**2.8**	**1.239**	**30.0**	**0.22**	**5.1**
	*62*	*34.4*	*2.2*	*1.5*	*1. 9*	*30.0*	*0.06*	*20.2*
	322							
i	**490**	**31.6**	**2.1**	**3.0**	**2.1**	**66.0**	**0.2**	**8.3**
	*70*	*34.6*	*5.1*	*2.4*	*4.3*	*35.0*	*0.07*	*25.1*
	560							
j	**95**	**31.6**	**0.6**	**1.9**	**0.6**	**59.0**	**0.09**	**45.8**
	*675*	*34.2*	*3.1*	*1.4*	*2.6*	*18.0*	*0.08*	*1.5*
	770							

The green and red numbers are the estimated values for the first and second vesicle clusters, respectively. The total numbers of vesicles per active zone (black numbers in parenthesis) are the sum of the bold and italic numbers.

### Fluorescence imaging

Individual neurons were viewed at their equator with a Nikon Eclipse TE 200 microscope through a Nikon 40x oil immersion objective (NA 1.30 and WD 0.22). Fluorescence FM 1–43 imaging was performed with excitation at 480 nm and emission at 535 nm. We used a cooled CCD camera (IMAGO, Till Vision, Germany) to acquire sequences of images of 640×480 pixels every 2 seconds with a 400 ms acquisition time per image. Images were stored digitally by using TILLvisION software. Only spots that were in focus were considered for our analysis. The focus was tested at the end of the experiment by z axis scanning. This allowed us to obtain volume estimates of the fluorescence spots, which were used to estimate the number of vesicles fused, as shown below. Some imaging was also made from the bottom of the dish to estimate the shapes of the release sites. Fluorescence measurements were made by linear interpolation after the fluorescence from a region containing no cell in each sequential image was subtracted from the intensity of the membrane in the corresponding image.

**Figure 7:**
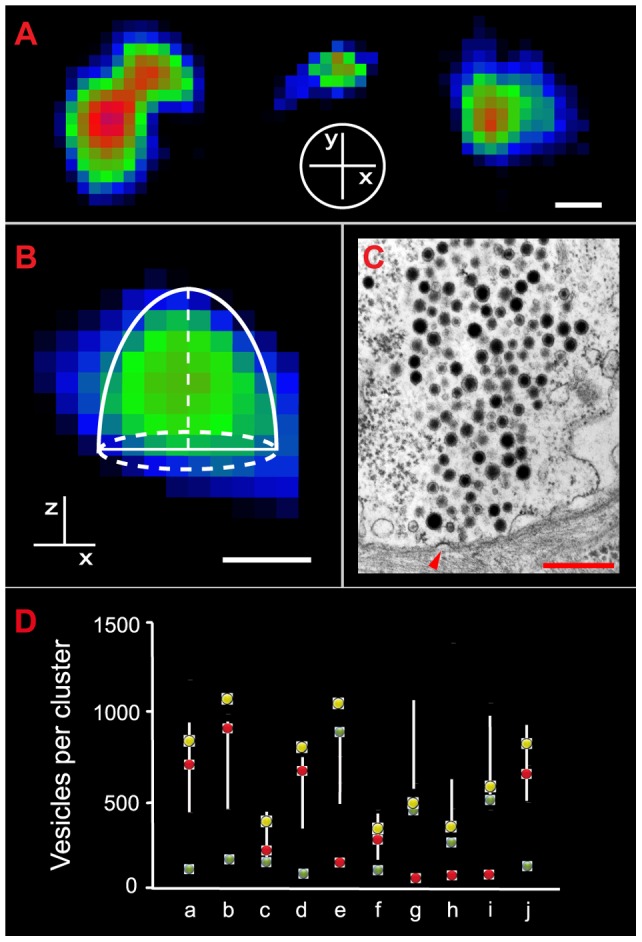
Number of vesicles fused in individual release sites. **A**. The release sites had nearly circular shapes as shown by FM1-43 fluorescent spots imaged at contact site of the plasma membrane with the glass bottom of the plate. The fluorescent spots on the left seem to be produced by two adjacent circular release sites, whereas the other two seem to be produced by single release sites. The axis inside the white circle indicate the plane of fluorescence with respect to the plasma membrane. **B**. Equatorial image of a fluorescent spot once the fluorescence level had reached a plateau. The horizontal line indicates the plasma membrane and therefore, the diameter of the release area. The vertical line indicates the depth of fluorescence inside the neuron and therefore the z projection (see text). The hemi-ellipsoid indicated by the white overlay is the approximation of the volume of the vesicle cluster loaded with FM1-43. For these estimates, the x and y lengths were considered as equal and were determined from the membrane front of the fluorescent spot. The volume was estimated as described in the methods. **C**. Electron micrograph of a vesicle cluster apposed to the plasma membrane in a neuron that had been stimulated at 20 Hz before fixation. The arrowhead points to a vesicle fixed at the moment of fusion. The profile of this vesicle cluster may be compared with the fluorescent spot shown in B. Scale bar  = 500 nm for all images. **D**. Number of vesicles fused per release site predicted by the model fittings to the plateau of the fluorescence kinetics (colored dots) and from the volumetric estimates of the active zones in combination with the vesicle density range (125–290 vesicles µm^−3^) obtained from electron micrographs (white lines). The green and red dots are the number of vesicles in the first and second clusters respectively; the yellow dots are the sum of the numbers of vesicles estimated for both vesicle clusters in each release site. Note that the small vesicle numbers were not resolved from the volumetric measurements.

### Quantitative analysis of exocytosis

The fluorescence kinetics was analyzed by assuming that fluorescence increases as FM1-43 stains vesicles as they fuse with the plasma membrane [25]. Our method consisted of measuring the cumulative FM1-43 fluorescence increase produced by the progressive exo/endocytosis of dense core vesicles in the clusters [20]. In our experience dense core vesicles that underwent fusion are not reintegrated to the releasable pool within the time course of our experiments and instead are transported back and packed into multivesicular bodies that later return to a recycling region near the Golgi apparatus [20], thus giving a following cluster the possibility to arrive at the same area of the plasma membrane. Therefore the gradual increase of fluorescence would be proportional to the number of dense core vesicles in the releasable pool. There is no evidence that dense core vesicles become fused again during short periods of time. However, if stained vesicles fuse again, the amount of fluorescence would not increase any further. Therefore, the estimate of the number of vesicles transported would be effectively estimated from the fluorescence increases.

**Figure 8:**
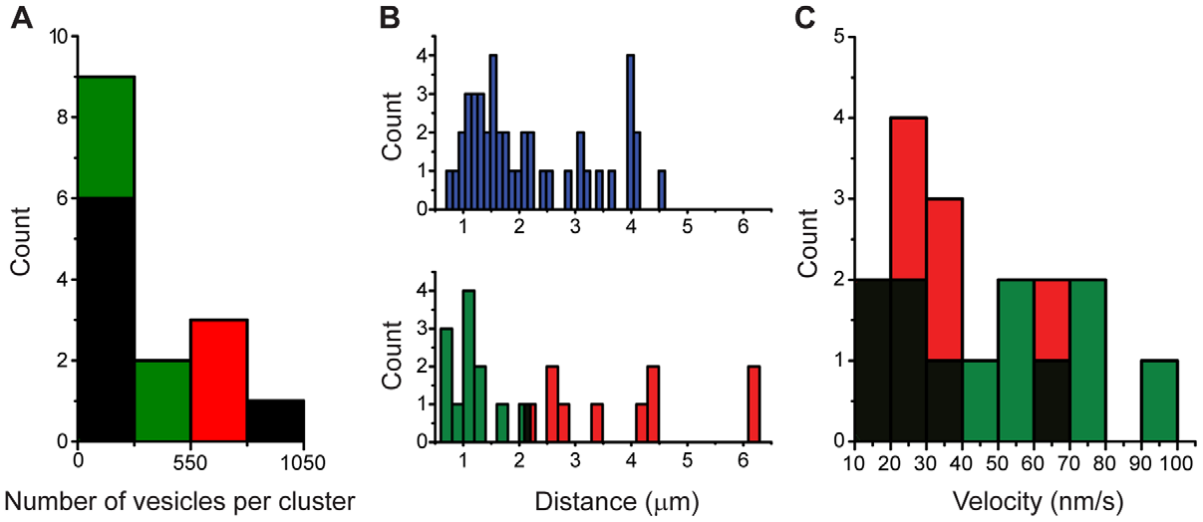
Distributions of the number of vesicles, traveling distances and velocities. **A**. the distributions of the number of vesicles contained in the first (green) and second (red) clusters overlapped. **B**. the distance distributions of the first and second clusters predicted by the model overlapped with that obtained from electron micrographs. **C**. there was overlapping also on the velocity distributions of the first and second clusters.

The fluorescence kinetics *F(t)* described in Eq. 1 is proportional to the number *n_0_* (Eq. 6) of vesicles fusing per time unit (see eq. 4) at an area *A* of the plasma membrane in the form:
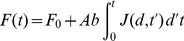
(1)Where *F_0_* is the basal fluorescence and *b* is a proportionality factor that relates the amount of fluorescence to the number of vesicles fusing in response to stimulation. This proportionality stems from the fact that the diameter (and therefore, the internal membrane surface) of the dense core vesicle population is considered constant (100 nm) and vesicles have similar and short fusion times [18]. Therefore, by keeping constant the FM1-43 concentration in the extracellular medium during the experiments we expect that the fusion of each individual vesicle produced a similar average fluorescence increase. Note that since fluorescence is the integral of the vesicle flow, the fluorescence changes are not linearly related to the number of vesicles fused.

**Figure 9:**
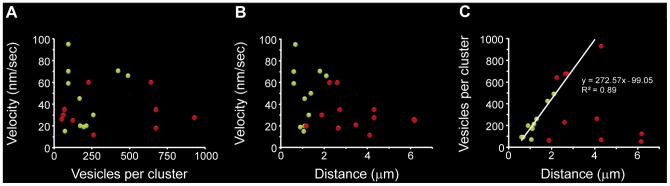
Relationships between the number of vesicles, traveling distances and velocities. **A**. the traveling velocity of the vesicle clusters had no correlation with the number of vesicles. **B**. The first clusters (green dots) had a wider range of velocities than the second clusters (red dots) and, the traveling velocity tended to decrease as the traveling distance became larger. **C**. While the number of vesicles in the first cluster (green dots) increased linearly with the distance, these variables did not correlate for the second clusters.

In writing Eq. (1) we have considered that the fluorescence kinetics also depend on their traveling distance *(d)*, considered as the distance from the center of mass of the cluster to the plasma membrane and the average velocity *(v)* of the vesicle cluster, that in turn renders the time-dependence (*t*) of the process. Therefore, the vesicle current flux *J (d, t)* is the main parameter to be evaluated. This is a good theoretical approximation, since as will be shown here, the long (even hundreds of seconds) duration of the FM1-43 fluorescence kinetics is consistent with an active vesicle transport from the resting positions of the vesicle clusters to the plasma membrane, but is 2–3 log units longer than the milisecond duration of individual vesicle fusions with the plasma membrane [18]. Since electron micrographs show that vesicles travel radially towards the plasma membrane, the term *J* is also consistent with a radial behavior of the vesicle current arriving at the membrane.

**Figure 10:**
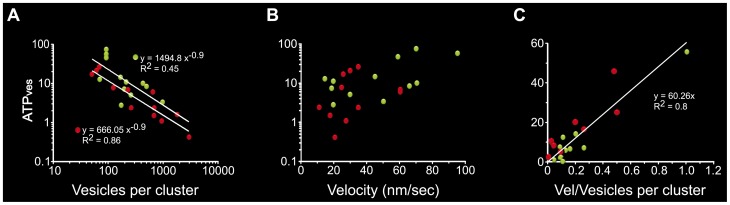
ATP-dependence of vesicle transport. Data from the first and second clusters are the green and red dots, respectively and the correlation coefficients for each data population are presented in each plot. **A**. The number of ATP molecules cleaved per vesicle fused (ATP_ves_) had inverse potency relationships with the number of vesicles per cluster. **B**. The ATP_ves_ values increased as the traveling velocity was larger. **C**. The ATP_ves_ values increased linearly as a function of the ratio of the velocity and the number of vesicles per cluster, suggesting that these variables were physiologically linked.

The simplest model that we could formulate to estimate the vesicle current *J (d, t)* considers the sum of forces per unit mass *fi* applied to the vesicle clusters according to Newton's law:
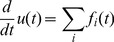
(2)where *u(t)* is the instantaneous velocity of a vesicle cluster.

As shown in Fig. 4a, we considered that the vesicle current density is influenced by four different forces (*f_f_, f_R_, f_el_* and *f_mot_*). The first force 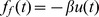 is the frictional force due to the resistance opposed by the medium to the vesicle motion. This force is proportional to the traveling velocity of the vesicles. The friction coefficient per unit of mass is 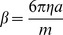, where 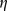 is the viscosity of the medium, *m* is the mass of the vesicle cluster, that may include mitochondria and endoplasmic reticulum, and a is the 50 nm radius of the individual vesicles. The term 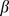 can be inferred from the predictions of force and velocity, as will be seen in Eq. (5).

**Figure 11:**
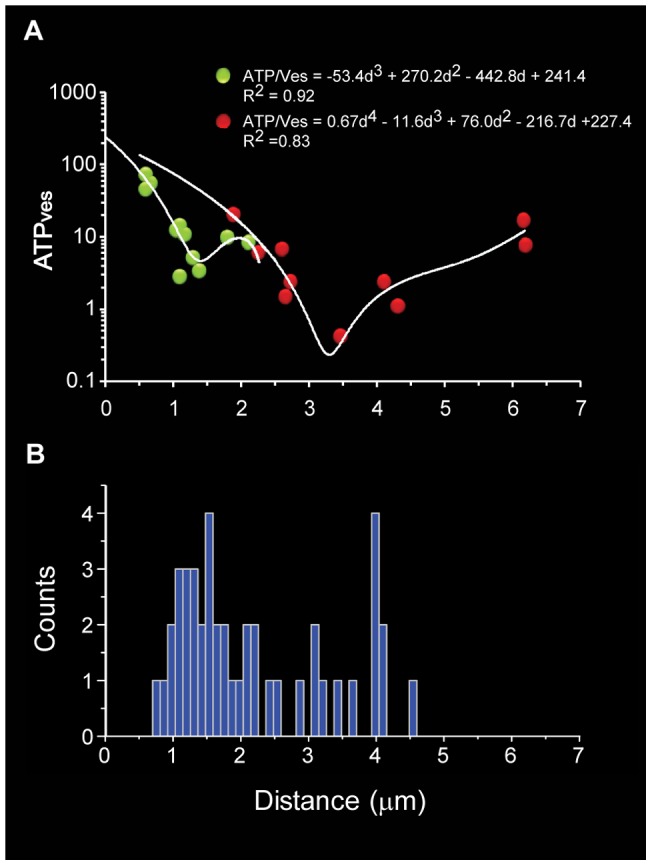
The distance-dependence of the ATP cost was bistabile. **A**. The ATP_ves_ values had a bistable relationship with the traveling distance. The equations describing the behavior of the first (green) and second clusters (red) are shown. The overall form of the curves was asymmetric, with ATP_ves_ values in the short distance range corresponding to the first clusters. The high energy barrier was at the intersection between data from both populations of vesicle clusters. **B**. The low energy distances of the curves in A correlate with the distance distribution of the clusters, measured form electron micrographs.

The second force is random *f_R_(t)*, produced by the thermal agitation of the medium. These two forces are responsible for the Brownian motion (confined diffusion) performed by the vesicles at a temperature T (20°C). The third and fourth forces are the elastic and the motor forces, 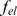 and 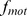, respectively, which act in combination. Where:
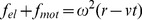
(3)


The elastic forces confine the vesicles in the cluster with a frequency
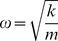
(4)that characterizes the magnitude of the force through its relationship with the effective elastic constant k of the cytoskeleton [26]–[27]. At rest, when the velocity v = *0*, the vesicle cluster mobilization is restricted by the elastic forces to an internal region of the neuron, defined here as an initial position *r*_0_,_i_, where i denotes the *i*_th_ vesicle. The motor forces are exerted by the molecular motors on the vesicle clusters upon electrical stimulation and produces their mobilization with an average constant velocity v along cytoskeletal filaments towards the plasma membrane [28], [29]. These forces along with the cargo imposed by the vesicles are the major contributors to the average constant velocity *v* of the vesicle transport [29].

The model formulated for *J* is based on Fick's law adapted to the current density of vesicles *J(r, t)* in the presence of the external forces:
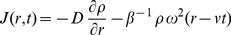
(5)Where *D* is the diffusion coefficient of the vesicles within the cytoplasm, 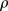
is the density of particles; which allows a numerical evaluation of Eqs. (1) and (3), as will be defined in equation (5), and r(t) is the position of the center of mass of the cluster at time t. Note that r = d when t = 0. The first term on the right-hand side of Eq. (4) is the constrained diffusion term incorporating implicitly *f_f_* and *f_R_*. The terms on the left hand side of equation 4 are again 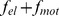.

The density of particles 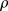 raises from the diffusion current defined in Eq. 5, and is is given by:
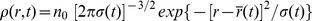
(6)where 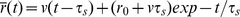 is the average position of the vesicle cluster, and 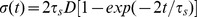 is the variance of the probability distribution describing to the Brownian motion of the cluster.

### Estimate of ATP consumption

We used the sum of the total non-dissipative forces per unit mass (in units of N/kg [ = ] m/s^2^), which are the motor and the elastic forces shown in eq. 3. The total force (in N) used then to move a vesicle cluster, taking into account every other cargo traveling with the cluster, such as mitochondria and other organelles, whose mass is m_tot_ (in kg) will be given by:
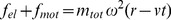
(7)


But harmonic components are related to the transport along the cytoskeletal structure, as has been defined in eq. 4^.^ Therefore, substitution of this relation into equation (7) we have:
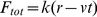
(8)


Since the intracellular environment is a highly dynamic heterogeneous medium, its viscoelastic properties are described by dynamic fields. In particular, K  =  K(r, t) where r is the position vector with respect to some coordinate frame. Since in this case r = d, the total work performed to move the vesicle cluster along a distance “d” is then:
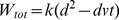
(9)


By definition, the change in free energy equals the ideal work (W_tot_) hence,
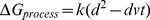
(10)


Due to vesicle cluster size heterogeneity, we expressed the energy related quantities in terms of unit vesicle. In doing so, we will be able to calculate the total energy cost of transporting one serotonin-containing vesicle. We then define the vesicle-specific free energy chage, ΔG_ves_ as,
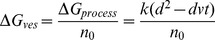
(11)


The change in free energy necessary to activate the molecular motors in such viscoelastic environment is supplied by ATP consumption. Since consumption of an ATP molecule typically releases an amount of energy ΔG_ATP_, the following relationship renders the number of ATP molecules used per vesicle fused (ATPves).
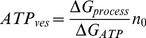
(12)


The ΔG_ATP_ value used was 5.4×10^−20^ Joules [30]. The ATP_ves_ (equation 12) and its functional dependence on the biophysical parameters required for their transport has been difficult to estimate directly in living neurons up to date. In the present case, the time was measured directly from the fluorescence kinetics; the distance and the number of vesicles were obtained from the model fittings to the fluorescence kinetics and tested independently from electron microscopy; the transport velocity and the space-dependent elastic constant K was determined by self-consistent methods from the fittings of the model to the fluorescence dynamics, as given in the subsection “Quantitative analysis of exocytosis” (equations 1–5).

### Curve fitting

For several parameters, pre-determined sets of values were used. For example, the intracellular diffusion coefficient was obtained from [23]; the velocities, and the traveling distances were within the ranges established from electron microscopy, and the numbers of vesicles were correlated with those estimated from a combination of electron microscopy and volumetric measurements of fluorescence, as will be shown below. The inclusion of experimentally tested values restricted the number of possible combinations of values estimated by the model.

Fitting of the data to the model was done manually. This was preferred over minimum square methods since sigmoidal functions with two crossovers become smoothed in abrupt parts of the curves that have strong contributions by the motor activity.

## Results

### Kinetics of exocytosis

Stimulation of individual neurons with 20 Hz trains delivered every 2 minutes produced spotted FM1-43 fluorescence increases at the neuronal surface, with each spot suggesting a the fusion of multiple dense core vesicles [3], [4]. In equatorial images of the neurons, the fluorescence per spot continued to increase over several minutes after stimulation (Fig. 1) with complex kinetics that as will be seen below could be fitted by two sigmoidals (see also Fig. 5) that suggested the arrival and fusion of an equal number of vesicle clusters. The formation of different equatorial spots in any one neuron had similar fluorescence kinetics, although their size and maximum fluorescence levels varied (Fig. 1C), thus suggesting that different amounts of vesicles were fused at each release site. As previously shown, these fluorescence increases did not occur in neurons stimulated at 1 Hz or at high frequency with calcium substituted for magnesium in the external solution [3].

### Ultrastructural basis of vesicle transport and exocytosis

Sections of five Retzius neurons that had been stimulated with 1 Hz trains had the already known [20] peripheral pool of dense core vesicle clusters (Fig. 2). However, when the distances between the center of these vesicle clusters and the plasma membrane were measured, the distribution of distances had two sub-populations, correlating with the two fluorescence increases seen with the 20 Hz stimulation protocol. The more peripheral subpopulation rested at 1.5±0.2 (SEM) µm from the plasma membrane (64% of the 44 clusters studied), and their proximity to it suggested that they would produce the exocytosis of the first sigmoidal fluorescence increase. Therefore, from now these will be called “first clusters”. The more internal subpopulation included 36% of the clusters and displayed a more scattered distribution (shown in Fig. 8B), with an average distance at 3.7±0.3 (SEM) µm from the plasma membrane. These will be named “second clusters” by supposing that their fusion with the plasma membrane would produce the second bulk of exocytosis.

By contrast, neurons that were fixed after 20 Hz stimulation showed vesicle clusters closely apposed to the plasma membrane (Figure 2 B and D), with omega-shaped figures indicating vesicle fusion [20]. In addition to these previous observations, we report here that after 20 Hz stimulation, the vesicle clusters that had arrived at the plasma membrane were near microtubules and mitochondria (Fig. 2A, C; Fig. 3), suggesting that they had been transported together with their energetic machinery.

That whole vesicle clusters are transported towards the plasma membrane upon 20 Hz stimulation was supported by the similarity of structures regardless of their positions inside the neurons or the stimulation protocol. In all cases, rows of dense core vesicles within the clusters were bound to individual microtubules through thin electrodense filaments (Fig. 3). Moreover vesicle clusters apposed to the plasma membrane kept their associations with the bundles of microtubules, which may be the rails for their centripetal transport (Fig. 2A, C).

The density of vesicles per µm^3^ in the clusters was estimated from electron micrographs, in which we measured 50–100 nm distances between the centers of contiguously aligned 100 nm dense core vesicles. These distances were similar for contiguous vesicles in a row or between vesicles in adjacent rows, thus rendering a density range of 296–125 vesicles per µm^−3^, respectively (see Table 1). These measurements are used below in combination with fluorescence measurements to confirm the number of vesicles fused per active zone.

As already shown [20], the perinuclear region contains another pool of vesicle clusters in the process of formation (Fig. 2A, B). Because of their apparent lack of maturation and the detection of only two sigmoidal fluorescence increases in response to electrical stimulation, the contribution of these vesicles to exocytosis is not expected to occur under our experimental conditions.

### Theoretical mechanism of the fluorescence increases

The data presented above are resumed in the scheme of Fig. 4 to explain the sigmoidal fluorescence kinetics, based on the following sequence of events: a) the slow initial fluorescence increase preceding the dynamic interval reflects exocytosis from vesicles that were near the plasma membrane at the onset of stimulation; b) the latency to reach the dynamic interval is proportional to the distance between the resting vesicle clusters and the plasma membrane and is inversely proportional to the average velocity of the vesicle transport, which in turn is affected by the number of vesicles in the cluster; c) the dynamic interval is proportional to the intracellular vesicle flow, which is in turn proportional to the average velocity of the vesicle transport, the number of vesicles, the release area, the ratio of vesicle fusion, and the effective friction of the intracellular medium (Eqs. 1 and 2). It is also worth noting that the slope of the dynamic interval also indicates the rate of vesicle fusion with the membrane. However, since the last steps of exocytosis once the vesicles have arrived at the plasma membrane do no depend directly on the active vesicle transport, we will not analyze this issue here; d) the maximum fluorescence value is proportional to the number of vesicles in the cluster and is reached when the releasable pool of vesicles has fused with the plasma membrane. This part of the hypothesis is supported by the data on the vesicle cycle described in the Methods section and also by the fact that a second vesicle cluster arrives at the same plasma membrane of the active zone. These possibilities were tested by fitting our data to sets of equations that consider motor, elastic and diffusion forces acting on vesicle clusters bound to the cytoskeleton (see Materials and Methods).

### Simulations of active zone fluorescence kinetics

The model reproduced the fluorescence kinetics of each individual active zone (n = 12), provided that two subsequent kinetics were adjusted with different parameter values, indicating that two consecutive vesicle clusters contributed to the overall fluorescence kinetics of each release site analyzed. However, the model failed to fit with the data when the contribution of the motors was eliminated from the simulations by cancelling their velocity in Eq. (3). Figure 5 shows the fluorescence kinetics of three fluorescent spots from different neurons, superimposed to model predictions obtained by adjusting the values of the traveling velocity (v), the number of vesicles in the cluster (*n_o_*), the distance (*d*) from the center of mass of the cluster to the plasma membrane, the elastic forces acting on the vesicles (*ω*) and a diffusion coefficient (D). All the values obtained for each release site can be seen in Table 1.

The fluorescence kinetics of cell “h” in Table 1 and plotted on the left of Fig. 5A displayed clearly both consecutive sigmoidal kinetics. The green and red traces are the predicted individual contributions of each successive vesicle cluster. In this case, the first step was produced by the fusion of 423 vesicles that had traveled 1.1 µm at 45 nms^−1^, while the second step was produced the fusion of only 52 vesicles that had traveled 4.3 µm at 27.5 nms^−1^ (see neuron “h” in Table 1). Note that when the velocity was zeroed as a way to eliminate the motor contribution in the equations, the kinetics became purely diffusive (blue lines in Fig. 5A). Two other examples of the fluorescence kinetics of two other release sites (“c” and “I” in Table 1) are also shown for comparison in Fig. 5A. The kinetics displayed by the active zone of cell “c” were different from each other. The number of vesicles in the second cluster (169) was nearly 5 times smaller than that of the first cluster (930). Moreover, the velocities were also different (see Figure legend and Table 1). Both subsequent kinetics obtained from cell “i”, had similar 30 nm/sec velocities and both clusters had closer traveling distances of 1.28 and 1.89 µm, respectively, but again, each cluster had a different number of vesicles (260 in the first and 62 in the second clusters).

Figure 5B shows the effects of changing the traveling distance (left), average traveling velocity (center) and number of vesicles per cluster (right) on the fluorescence kinetics of the second vesicle cluster of cell “c” in Fig. 5A. It may be seen that reducing the traveling distance reduced the latency to reach the dynamic range of the sigmoidal kinetics; increasing the velocity had a proportional increase in the slope of the sigmoidal fluorescence increase, and increasing the number of vesicles in the cluster increased the maximum fluorescence value. Opposite effects occurred when these values were reduced.

### Microtubule disruption eliminates the fluorescence increases

Inhibition of microtubule polymerization by addition of colchicine to the culture medium 30 min before stimulation prevented the sigmoidal fluorescence increases induced by electrical stimulation, without affecting the electrical properties of the nine neurons tested (Fig. 6). Five hundred seconds after the onset of stimulation the levels of fluorescence had increased only by 10% when compared to the 100–400% increases produced by a similar stimulation pattern in non-treated neurons. Again, data were well-fitted by the model when the contribution of molecular motors was eliminated by considering that v = 0 nms^−1^ (Fig. 6a).

### Number of vesicles fused per active zone

The number of vesicles fused per active zone was an essential estimate for our analysis. For this reason, apart from the values obtained from the model (Table 2), we made independent estimates by calculating the volume of release sites in different neurons from the areas of the fluorescent spots once the second kinetics had reached their plateau, and calculating the number of fused vesicles contained in them from the vesicle density of the clusters estimated from electron micrographs.

From images of individual fluorescence spots formed at the bottom of the plate we detected that the release surface areas of the plasma membrane are nearly circular (Figure 7). Therefore, the membrane front of the fluorescent spots in equatorial images indicated the diameter of the release area. Second, we assumed that the intracellular area of the equatorial fluorescent spot contained the information about the cluster size after the sequence of exo/endocytosis. As may be seen in Fig. 7, the equatorial fluorescent areas were well represented by hemi-ellipsoids and therefore, their volume was obtained as:
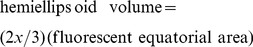
(13)with 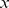 being the front of the fluorescent area and therefore its diameter.

For release sites larger than 1.5 µm we detected that the sources of error increase owing to geometrical and optical reasons, partly due to vesicle fusion in two adjacent release sites (Fig. 7A). Therefore, these cases were excluded from our analysis and we only used data from fusion areas with radiuses of 0.6–0.85 µm. In these conditions, the volume of the clusters ranged between 1.2 and 3.5 µm^3^ (see Table 2). As seen in Figure 7D and Table 2, the numbers of vesicles per active zone estimated by this method were similar to the number of vesicles in the clusters predicted by the model, provided that the cluster contained more than 100 vesicles. This result steams from the fact that volume measurements were made after the second sigmoidal fluorescence increase had reached a plateau and therefore represents the sum of fluorescence produced by two clusters. However, we consider that the fitting of the data justifies using the model predictions of the number of vesicles as the basis for the rest of our study.

### Estimates of the traveling distance and velocity

The traveling distance ranges of the first and second vesicle clusters predicted by the model were 0.6–2.1 µm and 1.9–6.2 µm, respectively and also fitted well with the ranges estimated by electron microscopy (Fig. 8B, Table 2). We also found that the transport velocity ranges of the vesicle clusters predicted by the model were similar for the first (15–95 nm/sec) and second (11–70 nm/sec) clusters. All these data are presented in Table 2. Moreover, consecutive vesicle clusters arriving at each release site did not have direct interactions, since their velocities had two- to four-fold non-correlated (R^2^≤0.4) variations. Therefore we did not carry out any further analysis in the possible interactions between successive clusters.

### Interactions between the number of vesicles, their traveling distance and velocity

The number of vesicles per cluster and their traveling velocity had correlation coefficients below 0.4 (Fig. 9A). There was also a low correlation between the traveling velocity and distance (Fig. 9B). However, as the distance was larger, the traveling velocity tended to decrease. The data shown in Figure 9B shows that the velocity range of the first clusters was larger (19–95 nm sec^−1^) than that of the second clusters (11.5–60 nm sec^−1^). Moreover, nine out of 11 of the second clusters travelled at low velocities of 11–35 nm sec^−1^, while 7 out of 11 second clusters traveled at larger velocities. Moreover, five of the first clusters travelled at velocities between 59–95 nm sec^−1^. These results had an interesting effect in the predictions of ATP consumption, as will be seen below.

As shown in Fig. 9C, the number of vesicles in the first cluster was linearly proportional to the traveling distance (R^2^ = 0.89) within the range from 0–2.2 µm. These data shows that as the clusters approach the plasma membrane they lost vesicles at a rate of 272 vesicles per µm^−1^. The intersection with the y axis indicates that in average 100 vesicles (99.5) arrive spontaneously at the membrane and this would be the average number of vesicles per release site producing the spontaneous release detected previously in these neurons [3]. The number of vesicles belonging to the second cluster had a larger dispersion without any correlation with the traveling distance (Fig. 9C).

### Estimate of ATP consumption per vesicle fused

The values of ATP_ves_ estimated from the work exerted by the molecular motors (see methods) ranged between 1 and 70. As shown in Figure 10A, the ATP_ves_ and the *n_o_* values were inversely proportional, and their behavior was described by a potency equation with a correlation coefficient R^2^ = 0.86 for the second cluster thus suggesting an increasing positive co-operativity in the motor performance as the cluster size was increased. Note that the correlation for the data of the first cluster was only R^2^ = 0.45, maybe due to the contribution of a second set of motors as vesicle clusters are closer to the plasma membrane (see discussion). The ATP_ves_ values increased in proportion with the traveling velocities, although the data distribution shown in Fig. 10B produced low correlation coefficients for the first (R^2^ = 0.40) and second clusters (R^2^ = 0.15). However, when the ATP_ves_ values were plotted as a function of the v/*n_o_* ratio, the data were linearly proportional, with the same correlation coefficients (R^2^ = 0.8) for the first and second clusters (Fig. 10C). This suggested that the number of vesicles and their traveling velocity were functionally linked.

### Bistabile relationship between the ATP consumption and the travelling distance

An interesting bistabile relationship was found between the ATP_ves_ values and the travelling distance of the vesicles belonging to the first and second clusters (Fig. 11A). A cubic polynomial equation fitted well with the data from the first cluster (R^2^ = 0.92), having a minimum value of 3–4 ATP molecules per vesicle at 1.4 µm from the plasma membrane. A quadric equation fitted with the data from the second cluster (R^2^ = 0.83), with a second minimum of 0.4 ATP_ves_ at a 3.3 µm distance from the plasma membrane. These minimum values correlated again with the peaks of the distance distributions of the vesicle clusters obtained from the electron micrographs (Fig. 11). The intersection of both data populations formed a high-energy barrier at 1.6–2.0 µm, with a requirement of 10–20 ATP_ves_.

## Discussion

Brief electrical stimulation triggers a microtubule-dependent and long-lasting somatic exocytosis by Retzius neurons. We developed a method for quantifying the biophysical variables of vesicle transport preceding somatic exocytosis of serotonin and its energy expenses. By combining electron microscopy, the kinetics of exocytosis and a mathematical model we predict that the kinetics of exocytosis in individual active zones depends on the number of vesicles in the cluster, their initial distance from the plasma membrane and their transport velocity. Based on the forces acting on the vesicles we also estimated the ATP expenses for each vesicle fused and showed that they depend on the ratio of the velocity and the number of vesicles in the cluster, and also that they have a bistable relationship with the traveling distance, with the low energy states correlating with the resting positions of the vesicle clusters. Altogether these data confirm that active vesicle transport is a necessary intermediate step of the excitation-secretion coupling.

### A possible mechanism for somatic exocytosis

The time course of somatic serotonin secretion obtained here is similar to that obtained by use of amperometric records from the soma of these same neurons after addition of a calcium ionophore [18] and also from the soma of dorsal Raphe neurons in culture, in which vesicles travel from the perinuclear region to the plasma membrane [4], [5]. This time course is also similar to that of somatic secretion of dopamine [21] and ATP in other neuron types [31, for review see ref. 2] and to that of cathecolamine secretion from chromaffin cells [32]. These data support that some of the mechanisms described here may be present in the soma of other neuron types. By contrast, the shorter 5 sec time course of extrasynaptic serotonin release detected from Raphe neurons [33] may reflect its release from dendrites and axonal varicosities in which vesicles rest at nanometer distances from the plasma membrane, possible reflecting a smaller scale version of the mechanism studied here.

The 500 ms duration of the stimulation trains is strikingly short when compared with the minute-lasting secretion kinetics they evoke. It is worth mentioning here that single train stimulation evokes mono-sigmoidal kinetics in these neurons such as those in Fig. 3, with time courses similar to those occurring here for the first sigmoidal component. These data, along with the blockade of exocytosis by colchicine support the possibility that the active transport of the vesicle clusters towards the plasma membrane uses microtubules as rails. From these data we propose that the active zone for somatic exocytosis of serotonin in Retzius neurons includes mobile assemblies of vesicle clusters with endoplasmic reticulum and mitochondria, which upon electrical stimulation use the microtubule bundles as rails for their transport towards the plasma membrane fusion sites. The coexistence of the endoplasmic reticulum and the mitochondria with the vesicles and the cytoskeleton may allow a local calcium wave amplification and propagation followed by the calcium-dependent activation of ATP production by the mitochondria [34] that are assembled in close association to the transport system. In this regard, it is worth noting that the ATP_ves_ values depend on the cargo, which is dominated by the large mass of the mitochondria and endoplasmic reticulum that are logarithmic units larger than the mass of the vesicles.

On the other hand, the long time course of exocytosis raises the question about how is it sustained for such long periods after stimulation has ended. Transmembrane calcium entry followed by calcium-induced calcium release triggers neuronal somatic secretion in neurons of vertebrates and invertebrates [2], [20], [21]. Therefore, we may predict that transmembrane calcium entry triggers a rapid exocytosis from vesicles near the plasma membrane, accompanied by a centripetal calcium wave feed by a stepwise calcium-induced calcium release mechanism through the concentric relays of smooth endoplasmic reticulum. The ultrastructural assemblies formed by smooth endoplasmic reticulum, mitochondria, vesicle clusters and microtubules may allow the calcium concentration increases to trigger the local synthesis of mitochondrial ATP necessary to activate the cytoskeleton-based transport of the whole assembly towards the plasma membrane. How exocytosis is maintained as the vesicles arrive at the membrane even minutes after stimulation ended is still an open question. A possible explanation is that the early 5HT exocytosis induces its further release through the activation of autoreceptors and further intracellular calcium release, as it happens in lung cells [35].

### Possible motors contributing to somatic secretion in Retzius neurons

The resting positions of the more peripheral vesicle clusters in Retzius neurons are similar to the thickness of the F-actin cortex adjacent to the plasma membrane of chromaffin cells [6], [11]–[13]. Therefore, distal vesicle clusters must enter the actin cortex approach the plasma membrane. The bistable nature of the ATP expenses in relation to the distance suggests that at distances longer than 2.0 µm transport is based purely on the tubulin-kinesin system, and the energy barrier may reflect the energy cost of the entrance of the cluster to the actin cortex. The second low energy state may occur once the cluster is stabile within the cortex. The last stage of migration within the active cortex may be complemented with an actin-miosin transport that propels the vesicle clusters towards the plasma membrane. The summed cost of the parallel use of the tubulin-kinesin and actin-myosin transport systems may explain the higher energy cost of the last stage of the vesicle trip. Calcium may also participate here by inducing structural changes of the actin in chromaffin cells [13], [36]. Moreover, the similarities of the average transport velocity ranges estimated here with those through the actin cortex of chromaffin cells suggests that both transport stages in Retzius neurons have similar velocities. That the velocity of tubulin-kinesin transport in our experiments is smaller than that of tubulin-kinesin transport in vitro [37] may be explained by the large cargo size imposed by the vesicle clusters and the non-saturating ATP conditions of our experiments.

### Significance for serotonergic communication

Serotonin regulates multiple behaviors in vertebrates and invertebrates, by acting from single channels to whole circuits [38]–[42]. A single 20 Hz train applied to Retzius neurons activates about 100 active zones per soma [3], [4] and we also have unpublished evidence indicating that the axons also secrete large amounts of serotonin. Both Retzius neurons in a ganglion receive common inputs and produce similar responses upon stimulation of sensory cells [43]. Therefore, by considering that 100–1000 somatic vesicles fuse per release site upon one single train, the amount of vesicles fused by the par of neurons in a ganglion may be several tens of thousands. Therefore, the large amounts of serotonin released through somatic and in general, extrasynaptic secretion may be the primary source to exert the diverse serotonergic modulatory actions. By contrast, in rodent fibers, the peak concentration of extracellular serotonin released extrasynaptically occurs 5 sec after stimulation [33]. This time course difference may be explained by the smaller distances between the vesicles and the plasma membrane in the dendrites and axonal varicosities of Raphe neurons [44], [45]. Although the release mechanism of Retzius and rodent neurons may be similar, the time course differences may reflect their differences in space scales and working temperature of each system.

### General significance

Microtubule-associated motors contribute to vesicle traffic preceding exocytosis in multiple organisms and functions, from unicellular to mammals and by doing so they also contribute to pathogenesis [46], immune responses [47], release of peptide hormones [48] and slime secretion [49]. For example, embryonic sea urchin cells have two types of motors contributing to vesicle transport. An immediate and fast motion is due to activation of myosin motors whereas the motion of vesicle pools located at longer distances from the plasma membrane is due to kinesin motors [8]. However, owing to the small sizes and fast secretory responses to stimulation in many cell types, the kinetics of these events are difficult to analyze directly. Therefore we expect that our study may serve to understand the forces acting on vesicle traffic-dependent exocytosis in other cell types.

## References

[1] De-Miguel FF, Trueta C (2005) Synaptic and extrasynaptic exocytosis of serotonin. Cell Mol Neurobiol 2: 297–312. 10.1007/s10571-005-3061-z PMC11529639 16047543

[2] Trueta C, De-Miguel FF (2012) Extrasynaptic exocytosis and its mechanisms: a source of molecules mediating volume transmission in the nervous system. Front Physio 3: 175. 10.3389/fphys.2012.00319 PMC3432928 22969726

[3] Trueta C, Méndez B, De-Miguel FF (2003) Somatic exocytosis of serotonin mediated by L-type calcium channels in cultured leech neurons. J Physiol 547: 405–416. 12562971 10.1113/jphysiol.2002.030684 PMC2342656

[4] Kaushalya SK, Desai R, Arumugam S, Ghosh H, Balaji J, et al (2008) Three-photon microscopy shows that somatic release can be a quantitatively significant component of serotonergic neurotransmission in the mammalian brain. J Neurosci Res 86: 3469–3480. 18709651 10.1002/jnr.21794

[5] Colgan LA, Putzier I, Levitan ES (2009) Activity-dependent vesicular monoamine transporter mediated depletion of the nucleus supports somatic release by serotonin neurons. J. Neurosci 16: 15878–1587. 10.1523/JNEUROSCI.4210-09.2009 PMC2796554 20016104

[6] Vitale ML, Seward EP, Trifaró JM (1995) Chromaffin cell cortical actin network dynamics control the size of the release-ready vesicle pool and the initial rate of exocytosis. Neuron 14: 353–363. 7857644 10.1016/0896-6273(95)90291-0

[7] Steyer JA, Horstmann H, Almers W (1997) Transport, docking and exocytosis of single secretory granules in live chromaffin cells. Nature 388: 474–478. 9242406 10.1038/41329

[8] Bi GQ, Morris RL, Liao G, Alderton JM, Scholey JM, et al (1997) Kinesin- and myosin-driven steps of vesicle recruitment for Ca+-regulated exocytosis. J Cell Biol 138: 999–1008. 9281579 10.1083/jcb.138.5.999 PMC2136755

[9] Oheim M, Stühmer W (2000) Tracking chromaffin granules on their way through the actin cortex. Eur. Biophys J 29: 67–89. 10.1007/s002490050253 10877017

[10] Rosé SD, Lejen T, Casaletti L, Larson RE, Pene TD, et al (2002) Molecular motors involved in chromaffin cell secretion. Ann N Y Acad Sci 971: 222–231. 12438122 10.1111/j.1749-6632.2002.tb04466.x

[11] Manneville JB, Etienne-Manneville S, Skehel P, Carter T, Ogden, et al (2003) Interaction of the actin cytoskeleton with microtubules regulates secretory organelle movement near the plasma membrane in human endothelial cells. J Cell Sci 116: 3927–3938. 12928328 10.1242/jcs.00672

[12] Neco P, Giner D, del Mar Francés M, Viniegra S, Gutierrez LM (2003) Differential participation of actin- and tubulin-based vesicle transport systems during secretion in bovine chromaffin cells. Eur J Neurosci 18: 733–1742. 12924999 10.1046/j.1460-9568.2003.02801.x

[13] Trifaró JM, Gasman S, Gutiérrez LM (2008) Cytoskeletal control of vesicle transport and exocytosis in chromaffin cells. Acta Physiol (Oxf) 192: 165–72. Review. 10.1111/j.1748-1716.2007.01808.x 18021329

[14] Fuxe K, Dalhstrom A, Hoistad M, Marcellino D, Jansson A, et al (2007) From the Golgi-Cajal mapping to the transmitter based characterization of the neuronal networks leading to two modes of brain communication: wiring and volume transmission. Brain Res Rev 55: 17–54. 17433836 10.1016/j.brainresrev.2007.02.009

[15] Fuxe K, Borroto-Escuela DO, Romero-Fernandez D, Diaz Cabiale Z, Rivera A, et al (2012) Extrasynaptic neurotransmission in the modulation of brain function. Focus on the striatal neuronal–glial networks Front Physio 3: 175. 10.3389/fphys.2012.00136 PMC3366473 22675301

[16] Coggeshall RE (1972) Autoradiographic and chemical localization of 5-hydroxytryptamine in identified neurons in the leech. Anat Rec 172: 489–498. 4536825 10.1002/ar.1091720303

[17] Kuffler DP, Nicholls J, Drapeau PJ (1987) Transmitter localization and vesicle turnover at a serotoninergic synapse between identified leech neurons in culture. J Comp Neurol 22: 516–526. 10.1002/cne.902560404 2435767

[18] Bruns D, Riedel D, Klingauf J, Jahn R (2000) Quantal release of serotonin. Neuron 28: 205–220. 11086995 10.1016/s0896-6273(00)00097-0

[19] Trueta C, Sánchez-Armas S, Morales MA, De-Miguel FF (2004) Calcium-Induced Calcium Release Contributes to Somatic exocytosis of Serotonin in Leech Retzius Neurons. J Physiol 547: 309–316. 10.1002/neu.20055 15389693

[20] Trueta C, Kuffler DP, De-Miguel FF (2012) Cycling of dense core vesicles involved in somatic exocytosis of serotonin by leech neurons. Front Physio 3: 175. 10.3389/fphys.2012.00175 PMC3368391 22685436

[21] Patel J-C, Witkovsky P, Avshalumov MV, Rice ME (2009) Mobilization of calcium from intracellular stores facilitates somatodendritic dopamine release. J Neurosci 29: 6568–6579. 19458227 10.1523/JNEUROSCI.0181-09.2009 PMC2892889

[22] Betz WJ, Bewick GS, Ridge RM (1992) Intracellular movements of fluorescently labeled synaptic vesicles in frog motor nerve terminals during nerve stimulation. Neuron 9: 805–813. 1418996 10.1016/0896-6273(92)90235-6

[23] Siddall ME, Trontelj P, Utevsky SY, Nkamany M, Macdonald KS (2007) Diverse molecular data demonstrate that commercially available medicinal leeches are not Hirudo medicinalis. Proc. R. Soc. B 274: 1481–1487. 10.1098/rspb.2007.0248 PMC2176162 17426015

[24] Dietzel ID, Drapeau P, Nicholls JG (1986) Voltage dependence of 5-hydroxytryptamine release at a synapse between identified leech neurones in culture. J Physiol 372: 191–205. 3723408 10.1113/jphysiol.1986.sp016004 PMC1192758

[25] Betz WJ, Bewick GS (1993) Optical monitoring of transmitter release and synaptic vesicle recycling at the frog neuromuscular junction. J Physiol 460: 287–309. 8387585 10.1113/jphysiol.1993.sp019472 PMC1175214

[26] Santamaría-Holek I, Rubi JM (2006) Finite-size effects in microrheology. J Chem Phys 125: 64907. 16942312 10.1063/1.2241190

[27] Santamaría-Holek I, Rubi JM, Gadomski A (2007) Thermokinetic Approach of Single Particles and Clusters Involving Anomalous Diffusion under Viscoelastic Response. J Phys Chem B 111: 2293–2298. 17291031 10.1021/jp0675375

[28] Schnitzer MJ, Block SM (1997) Kinesin hydrolyses one ATP per 8-nm step. Nature 388: 386–390. 9237757 10.1038/41111

[29] Svoboda K, Block SM (1994) Force and velocity measured for single kinesin molecules. Cell 77: 773–784. 8205624 10.1016/0092-8674(94)90060-4

[30] Alberty RA, Goldberg RN (1992) Biochemistry (1992) Standard thermodynamic formation properties for the adenosine 5′-triphosphate series. Biochem 3: 10610–10615. 10.1021/bi00158a025 1420176

[31] Zhang X, Chen Y, Wang C, Huang LY (2007) Neuronal somatic ATP release triggers neuron satellite glial cell communication in dorsal root ganglia. Proc Natl Acad Sci U S A 104: 9864–9869. 17525149 10.1073/pnas.0611048104 PMC1887586

[32] Chow RH, Klingauf J, Heinemann C, Zucker RS, Neher E (1996) Mechanisms determining the time course of secretion in neuroendocrine cells. Neuron 16: 369–376. 8789951 10.1016/s0896-6273(00)80054-9

[33] Bunin MA, Wightman RM (1998) Quantitative evaluation of 5-hydroxytryptamine (serotonin) neuronal release and uptake: an investigation of extrasynaptic transmission. J Neurosci 18: 4854–4860. 9634551 10.1523/JNEUROSCI.18-13-04854.1998 PMC6792557

[34] Denton RM, Mckormack JG, Edgell NJ (1990) Role of calcium ions regulation of mammalian intramitochondrial metabolism. Physiol Rev 70: 391–425. 2157230 10.1152/physrev.1990.70.2.391

[35] Fu XW, Nurse CA, Wong V, Cutz E (2002) Hypoxia-induced secretion of serotonin from intact pulmonary neuroepithelial bodies in neonatal rabbit. J Physiol 539: 503–510. 11882682 10.1113/jphysiol.2001.013071 PMC2290169

[36] Trifaró JM, Gasman S, Gutiérrez LM (2008) Cytoskeletal control of vesicle transport and exocytosis in chromaffin cells. Acta Physiol (Oxf) 192: 165–172. 18021329 10.1111/j.1748-1716.2007.01808.x

[37] Visscher K, Schnitzer MJ, Block SM (1999) Single kinesin molecules studied with a molecular force clamp. Nature 400: 184–89. 10408448 10.1038/22146

[38] Weiger WA (1997) Serotonergic modulation of behaviour: a phylogenetic overview. Biological Reviews of the Cambridge Philosophical Society 72: 61–95. 9116165 10.1017/s0006323196004975

[39] Raleigh MJ, McGuire MT, Brammer GL, Pollack DB, Yuwiler A (1991) Serotonergic mechanisms promote dominance acquisition in adult male vervet monkeys. Brain Research 559: 181–190. 1794096 10.1016/0006-8993(91)90001-c

[40] Kravitz EA (2000) Serotonin and aggression: insights gained from a lobster model system and speculations on the role of amine neurons in a complex behavior. J Comp Phys A 186: 221–238. 10.1007/s003590050423 10757238

[41] Alekseyenko OV, Lee C, Kravitz EA (2010) Targeted manipulation of serotonergic neurotransmission affects the escalation of aggression in adult male Drosophila melanogaster. PLoS One 5: e10806. 20520823 10.1371/journal.pone.0010806 PMC2875409

[42] Willard AL (1981) Effects of serotonin on the generation of the motor program for swimming by the medicinal leech. J Neurosci 1: 936–944. 7288474 10.1523/JNEUROSCI.01-09-00936.1981 PMC6564106

[43] Velázquez-Ulloa N, Blackshaw SE, Szczupak L, Trueta C, Garcia E, et al (2003) Convergence of mechanosensory inputs onto neuromodulatory serotonergic neurons in the leech. J Neurobiol 54: 604–617. 12555272 10.1002/neu.10184

[44] Chazal G, Ralston JJI (1987) Serotonin-containing structures in the nucleus raphe dorsalis of the cat: an ultrastructural analysis of dendrites, presynaptic dendrites, and axon terminals. J Comp Neurology 259: 317–329. 10.1002/cne.902590302 3294934

[45] Moukhles H, Bosler O, Bolam JP, Vallee A, Umbriaco D, et al (1997) Quantitative and morphometric data indicate precise cellular interactions between serotonin terminals and postsynaptic targets in rat substantia nigra. Neuroscience 76: 1159–1171. 9027876 10.1016/s0306-4522(96)00452-6

[46] Henry T, Couillault C, Rockenfeller P, Boucrot E, Dumont A, et al (2006) The Salmonella effector protein PipB2 is a linker for kinesin-1. Proc Natl Acad Sci U S A 103: 13497–13502. 16938850 10.1073/pnas.0605443103 PMC1569191

[47] Stinchcombe JC, Majorovits E, Bossi G, Fuller S, Griffiths GM (2006) Centrosome polarization delivers secretory granules to the immunological synapse. Nature 443: 462–465. 17006514 10.1038/nature05071

[48] Park JJ, Cawley NX, Loh YP (2008) Carboxypeptidase E cytoplasmic tail-driven vesicle transport is key for activity-dependent secretion of peptide hormones. Mol Endocrinol 22: 989–1005. 18202146 10.1210/me.2007-0473 PMC2276472

[49] Yu R, Kaiser D (2007) Gliding motility and polarized slime secretion. Mol Microbiol 63: 454–467. 17176257 10.1111/j.1365-2958.2006.05536.x

