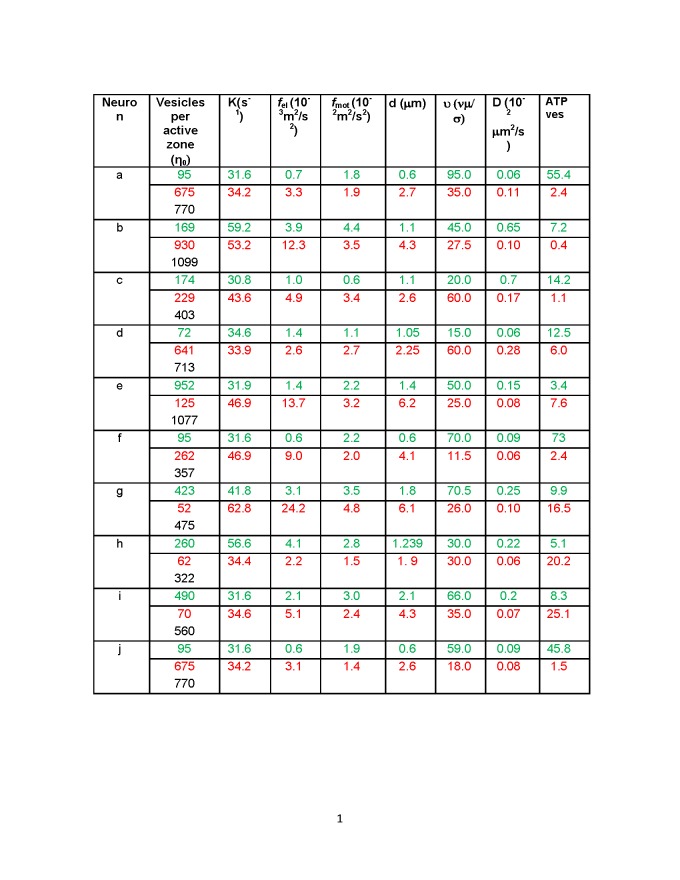# Correction: Biophysics of Active Vesicle Transport, an Intermediate Step That Couples Excitation and Exocytosis of Serotonin in the Neuronal Soma

**DOI:** 10.1371/annotation/b72afb21-407c-46e9-9870-7c59ab9e582c

**Published:** 2012-11-14

**Authors:** Francisco F. De-Miguel, Iván Santamaría-Holek, Paula Noguez, Carlos Bustos, Enrique Hernández-Lemus, J. Miguel Rubí

There was a formatting error in Table 2. The correct Table 2 can be viewed here: 

**Figure:**